# Management of delusions of parasitosis: an interview with experts in psychodermatology

**DOI:** 10.1097/JW9.0000000000000035

**Published:** 2022-07-08

**Authors:** Nicholas Brownstone, Josie Howard, John Koo

**Affiliations:** a Department of Dermatology, University of California, San Francisco, San Francisco, CA.

**Keywords:** delusions of parasitosis, delusional infestation, morgellons disease, psychodermatology

## Abstract

Delusions of parasitosis (DOP), which is also called Morgellons disease or delusional infestation, can be one of the most challenging clinical encounters in a dermatologist’s practice. One reason for this is lack of education during dermatology residency and a paucity of resources for the practicing dermatologist such as specialized psychodermatology clinics for these patients. To help close this knowledge gap, an interview was conducted with 3 experts in the field of psychodermatology and their responses were recorded and edited with goal of improving care for patients suffering from DOP by educating the busy, practicing dermatologist. The experts discussed many topics regarding DOP including the difference between primary and secondary DOP, how to build a good rapport with DOP patients, why the condition is seen mostly in older woman, and which medications are effective for treatment. The interview ends with a few high-yield practical treatment tips.

**What is known about this subject in regard to women and their families?**

- Primary DI is most frequently seen in older (postmenopausal) women.

**What is the take home message from this article for women and their families?**

- While, more data and research are needed on the topic of estrogen dysregulation and delusions of parasitosis, this article presents some evidence regarding postmenopausal estrogen dysregulation as an etiology for delusions of parasitosis.

## Introduction

Delusions of parasitosis (DOP), which is also called Morgellons disease or delusional infestation (DI), can be one of the most difficult clinical encounters in a dermatologist’s practice.^1^ This issue is exacerbated by the fact that few dermatology residency training programs offer formal training in psychodermatology, no official psychodermatology fellowship exists and there is a paucity of resources for the practicing dermatologist such as specialized clinics for these patients.^2^

As a negative result of this lack of education, many providers openly refer to these patients as “psychodermatology patients” or “delusional patients.” In the authors’ collective experience, this often damages the rapport between patient and provider.^3^ Learning how to talk to delusional patients and respect their ideation is almost necessary to deliver proper care to this patient population. This is true because these patients have usually seen many providers in the past who have dismissed their symptoms and have not respected their ideation which usually causes a contentious encounter in which the patient does not soon forget. The goal of this manuscript is to make the busy, practicing dermatologist more comfortable in treating DOP with advice from experts in the field.

## Methods

An interview was conducted among 3 experts in the field of psychodermatology and their responses were recorded below (N.B. is a dermatology fellow with 2 years of training in psychodermatology, J.H. is a psychiatrist specializing in psychodermatological issues and J.K. is a board-certified dermatologist/psychiatrist and director of the University of California, San Francisco Psychodermatology Clinic). Questions were specifically curated to maximally benefit the practicing dermatologist.

## Results

### Diagnosis

**Question:** What is the difference between primary and secondary DOP/DI, and why is it important to make the distinction?

**Answer:** DI has historically been called DOP to describe the fixed false belief that one is infested with parasites and more recently (in the past few decades) has been called Morgellons disease to describe the false belief that one has fibers or other foreign material infesting the skin.^4^ In Primary DI, a patient’s symptoms occur spontaneously in the absence of other dermatological conditions and is not secondary to another medical or psychiatric condition. In the authors’ collective experience, primary DI is most frequently seen in older (postmenopausal) women, which is also corroborated in the medical literature.^5^ Secondary DI refers to cases in which the delusions and/or hallucinations occur as a result of another underlying medical or psychiatric problem, most often substance use disorder or medication/alcohol withdrawal and is best treated by a psychiatrist.^6^

The distinction between primary and secondary DI is critical in guiding treatment planning and disposition. Although both groups most often present to the dermatology clinic, only primary DI is most appropriately treated in the dermatologist’s office. Moreover, a diagnosis of primary DI can only be made after excluding all other possible causes including primary skin infections (e.g., scabies, mites), underlying neurologic disease (head injury, dementia, vitamin B12 deficiency), medical conditions (e.g., hyperthyroidism, liver disease), psychiatric conditions (e.g., schizophrenia), medication side effects (dopamine agonists), or substance use/withdrawal (amphetamines, cocaine, alcohol).^7^ Finally, underlying psychiatric disorders such as schizophrenia, must also be excluded.^8^ Ideally, this is often best achieved through evaluation by other providers, including medicine, neurology, and psychiatry. However, this type of thorough evaluation may not always be possible, especially if the patient objects to an evaluation by a psychiatrist or neurologist.^9^ These patients often believe that acceptance of this type of referral is contrary to their belief system that the problem is caused by parasites or other foreign material.

**Question:** Are people who are very distressed about parasites all delusional?

**Answer:** Not every patient who presents with a chief complaint of “parasites” or “foreign material coming out of the skin” is delusional. By definition, a delusion is an unshakable belief in something that is untrue, and being “delusional” requires that the patient is absolutely fixed on their false belief and either unable or unwilling to consider any other explanations outside of their delusional ideation.^10^ Although a large proportion of these patients are delusional, there is also a significant proportion who have some degree of flexibility in their thinking and though they believe they are infested with parasites, they are open to entertaining alternative explanations for their experience. They may also sincerely tell the provider that they are primarily interested in getting rid of this problem and that goal is more important to them than discovering the cause of the problem. So, the clinical manifestation exists on a spectrum with this latter group consisting of people who still have some capability for flexible thinking and open mindedness regarding their “parasites” or “foreign material.”^11^ Distinguishing between patients who are truly delusional and those who may simply have an overvalued idea is critical as this will help determine the best approach the dermatologist should take towards speaking with the patient. If a patient has some flexibility in their thought process, the dermatologist can begin to gently introduce other causes for their symptoms and experience. It is imperative that the dermatologist first establish rapport and trust with the patient, validating their experience (although not their specific ideation) and emphasizing their common goal of decreasing the patient’s suffering. In the author’s experience, the window of opportunity to take advantage of the patient’s flexibility regarding causality of their experience is often small and may be lost if there is any hint of opposition, antagonism, or dismissal in the physician-patient relationship. If the patient is indeed delusional, the dermatologist needs to be extremely diplomatic. Clinically, the senior author (JK) has observed a trend in which patients seem to become more delusional the longer they go untreated with an antipsychotic medication or an anti-Tourette’s medication such as pimozide (which is effective in treating DI). Therefore, it is important that treatment is initiated as quickly as possible, either by a dermatologist or a psychiatrist, balancing the need for rapport with the potential risk of worsening symptoms.

**Question:** Why is this condition overwhelming seen in older women?

**Answer:** Primary DI, which is the most appropriate use of the term according to DSM- 5, is overwhelmingly seen in older woman.^5^ This simple and consistent observation suggests that DI may arise from an organic cause.^12^ With functional and brain imaging data, there is a theory proposed in Europe to explain this observation.^13^ The theory states that DI, like other psychotic disorders, such as schizophrenia, involves the presence of an excess of dopamine in the synaptic space. In women, as opposed to men, there is a predominance of a system called dopamine transport system (DAT) which keeps the dopamine level in a normal range. Unfortunately, this system may be dependent on estrogen levels for proper functioning and estrogen levels decrease with age. It is postulated that because of estrogen dysregulation leading to an increase in dopamine, psychotic disorders, such as DI, are more frequently observed in older women as opposed to older men. This also could explain why Parkinson’s Disease more frequently occurs in older men rather than women. Anecdotally, when these women with DI are successfully treated with an antipsychotic agent, some of them will develop pseudoparkinsonism as a side effect. In turn, when men with Parkinson’s disease are successfully treated with a dopamine agonist, some of them will develop DI. More data and research are needed on the topic of estrogen dysregulation and delusion of parasitosis before any formal conclusions can be made.

### Differential diagnosis

**Question:** What is the differential for a secondary cause of DI?

**Answer:** The differential diagnosis of a secondary cause of DI includes: illicit drug use (especially cocaine, narcotics, or amphetamines), primary psychiatric disorders (schizophrenia, major depressive disorder with psychotic features), traumatic brain injury and dementia.^14^

**Question:** What work up is recommended for possible DI?

**Answer:** The suggested laboratory examinations include CBC with differential, serum electrolytes, liver function tests, thyroid function tests, serum calcium, blood glucose, serum creatinine, blood urea nitrogen, Vitamin B12, urinalysis, toxicology screen, HIV, Hepatitis C, and RPR for Syphilis.^15^

The most important tests are thyroid function tests, Vitamin B12 and toxicology screen.

### Interpersonal connection

**Question:** How do you explain the etiology of this condition to the patient?/ How do you handle a patient who only is interested in getting validation that this is due to a parasite?

**Answer:** If the patient is delusional or close to being delusional, it is important to not confront the patient regarding the fact that the patient’s ideation is wrong because that can easily lead to an antagonistic relationship and damage the therapeutic rapport. Focusing on the etiology is unlikely to yield therapeutic success as these patients often maintain an obsessive fixation on investigating their complaints. The authors recommend that the dermatologist should work toward helping to shift the patient’s attention away from their intense focus on the exact etiology of this condition and refocus that attention onto the shared desire for a treatment, which will decrease the patient’s misery and suffering. One such approach may be to explicitly tell the patient that “nobody really knows what really causes DI or Morgellons disease.” In other words, rather than trying to explain the etiology, the emphasis is on trying to shift the patient’s attention from etiology to treatment. If the patient still insists on doing more investigations and is unable to focus on treatment, the authors do not discourage the patient from this but rather encourage the patient to see other professionals who are more suited for further investigation. These specialists include parasitologists, entomologists, or infectious disease specialists but the dermatologist should encourage them to come back for treatment if none of these other specialists could find an answer and the patient is still suffering.^16^ Moreover, reminding the patient that treatment with something that has empirically and repeatedly proven effective for patients with similar experiences need not preclude their continued investigation, but that the 2 may coexist. This is an “open door policy” even if the patient is not ready to accept treatment, they are welcome back in the office anytime to discuss their symptoms. However, the collective experience of the authors is that the patient is usually willingly to move on to treatment especially if the dermatologist explicitly and proactively reminds the patient how miserable their current situation is and why it is so important to get out of this current situation as soon as possible(even if the exact cause is still mysterious). Sometimes it is useful to remind the patient that in the practice of medicine, physicians successfully treat patients all the time for conditions where an exact etiology is not known. Examples of this situation range from “essential hypertension” to many conditions in dermatology such vitiligo, alopecia areata, lichen planus and pityriasis rubra pilaris.

**Question:** How can you maximize the chance of having a good connection/rapport?

**Answer:** It is not unusual for DI patients to be apprehensive and ambivalent when they are meeting a new dermatologist for the first time because they may have had negative experiences with other dermatology providers or other physicians who have shown no interest in their situation, who may have rushed them out of their office and who may have dismissed their symptoms. Therefore, it is important to enter the room with enthusiasm and positive energy. Knowing that these patients may have a fear of abandonment by the provider based on previous experiences, the authors at times explicitly tell the patient that “I will never give up on you, if you don’t give up on me.” In a rare instance, where the patient remains negative and pessimistic despite the above deliberate efforts, then it may be critical to remind the patient that he or she is meeting the dermatologist for the very first time and reassure the patient that the previous negative experience will not be repeated. However, it has been the authors’ collective experience that most patients do not persist in negativity if the above approach is taken where the new dermatologist appears to be actively engaged and eager to help.^17^

### Treatment

**Question:** What are some of the ways to get the patient to accept antipsychotic treatment?

**Answer:** One way to get the patient to accept antipsychotic treatment is to offer a medication that has no US Food and Drug Administration (FDA) psychiatric indication because DI patients are universally opposed to taking medications involving mental illness. In the US, pimozide has no FDA indication for any type of psychiatric disorder.^18^ Therefore, the provider can honestly tell the patient that the medication pimozide being offered (pimozide) is only FDA indicated for Tourette’s syndrome (a neurological disease) which has nothing to do with the patient’s condition; pimozide is used empirically in a “trial and error” approach as explained to the patient.

Another way to introduce the use of an antipsychotic is to explain that medications are often used for indications for which they were not originally developed. Aspirin, for instance, was developed as a pain reliever, yet is effective for cardiac prevention. Prescribing aspirin to someone for cardiac prevention does not imply that the prescriber thinks the patient has pain, just as prescribing an antipsychotic to someone with this constellation of symptoms does not mean that the doctor thinks the patient has schizophrenia.

**Question:** What are the major pros and cons of medications that are used to treat DI?

**Answer:** While second-generation antipsychotics, such as risperidone, carry a lower risk for tardive dyskinesia (TD), they are associated with a higher risk of metabolic disturbance.^19^ Perhaps an even more important practical consideration is patient compliance. The authors have found that antipsychotics are often rejected altogether by the patient because they all have US FDA psychiatric indications. Given the paramount importance of establishing trust and rapport, and the difficulty of earning back the patient’s trust if it is fractured, starting with a recommendation for a second-generation antipsychotic also carries with it the risk that the patient’s trust will be lost, as well as a crucial treatment opportunity.

Because pimozide’s primary indication is non-psychiatric, namely for Tourette’s Syndrome, we find that pimozide is often more acceptable to these patients.

**Question:** What is the dosing strategy when these medications are used?

**Fig. 1.**
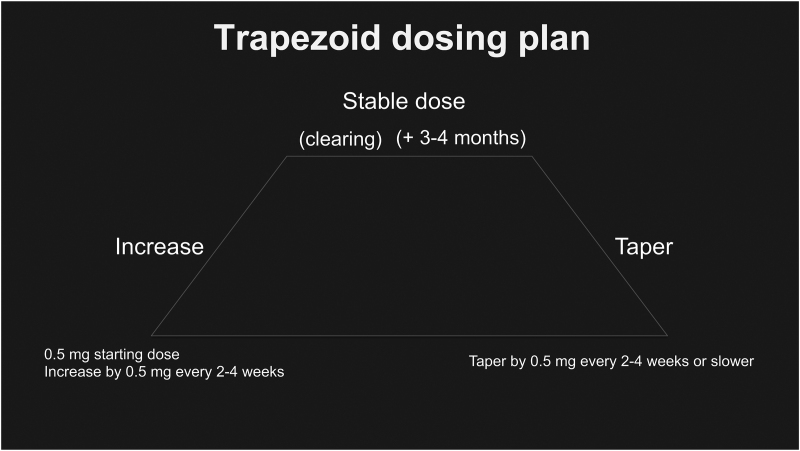
Trapezoid dosing regimen for pimozide and risperidone.

**Answer:** The dosing strategy resembles a trapezoid figure (Fig. 1). Referring to pimozide or risperidone, either medication is usually started with a very low dose of 0.5 mg to 1 mg per day. Then the dose is increased not faster than 0.5 mg increments every 2-4 weeks until an efficacious dose is reached for the particular patient in question. This dose is continued for 3-4 months before any further adjustments should be made, such as decreasing the dose to taper it off. There is no absolute maximum dose however, most patients show clinical response by the time the dose of 3 mg per day is reached. The senior author (JK), in his more than 3 decades of practice, had only 1 case who required more than 10 mg per day of pimozide to successfully treat the delusion. Even this patient’s symptoms were eventually managed by 3 mg of pimozide per day. Patient usually do not have recurrences if the medication is tapered properly but if the patient experiences a recurrence, the same trapezoid dosing method can be used to treat these symptoms effectively. Common side effects include minor stiffness or restlessness. While TD is extremely rare with the use of pimozide in the treatment of DI, practitioners should be aware that TD now has 2 FDA-approved medications for treatment.^20^

### Logistical tips

**Question:** What are some tips and tricks that can be used in treating DOP?

#### Answers:

##### Scheduling patients at end of clinic

Do not have these patients come in the middle of clinic because that can lead to disruption of normal patient flow. If the patient unexpectedly appears in the middle of the clinic for the first time, see below for temporizing strategies.

Since these patients likely do not need lengthy procedures, as long as the dermatologist is willing to be in the clinic beyond closing time, there is no negative financial impact because there is no increased overhead. There may even be some extra financial gain since these visits can be charged based on time. The dermatologist should use their discretion regarding safety if they choose to see these patients after office staff has left clinic. Of note, these patients may require a KOH prep or biopsy procedure to rule out a true parasitic infection or true dermatosis if the clinical scenario warrants such testing.

##### Temporizing strategy: Referring to them as VIP

If a DI patient unexpectedly appears as a new patient in the middle of a busy clinic, after spending 5-10 minutes intensely listening to the patient’s complaint, the provider can use some “temporizing” strategies by first asking the patient to return another day at the end of the clinic.^21^ The provider can also reassure the patient that he or she is taking their concerns seriously by telling the patient that she or he is “VIP” patient and therefore deserves more time and attention for such an important and complex situation. This way, the provider can manage new DI situations without disrupting the usual patient flow.

It is best not to use any psychiatric terms such as “delusion,” “psychosis,” or even “psychodermatological” in the notes because the patient can always access their medical records. Instead, it is better to use a neutral term that is not offensive to the patient such as “cutaneous dysesthesia” or “formication.” If the patient is at the delusional end of the spectrum, in order to communicate to other healthcare providers about the psychiatric nature of the case, the authors recommend using verbatim quotes of what the patient actually says. In this case a bizarre expression from the patient is useful because it clearly demonstrates to another provider that the patient is delusional without having to explicitly state it in the medical record. If a patient does request their records, they are unlikely to take offense at reading an exact quote that was said by the patient; instead they often appreciate the fact that the provider was paying so much detailed attention to their concerns. Any other provider who sees the following quote in the chart, “patient reports seeing a parasite with 20 legs and 15 eyes emerge from her skin and fly around the room before bed” will obviously know the patient is suffering from delusions.

##### Preparing specimens using a glass slide and clear tape

DI Patients frequently bring in specimens in many different ways, such as floating in dirty water.^22^ One way to handle this situation, while at the same time keeping the patient satisfied, is to let the patient know that specimens need to be examined under the microscope in order to be properly be evaluated. Then, give the patient several microscope glass slides without cover slips and ask the patient to prepare the specimens and bring them back to the next visit. Most importantly, instruct the patient to use absolutely clear tape such as packing tape instead of the usual “scotch tape” that is opaque. If opaque or matted tape is used by accident, specimens cannot be seen clearly. By giving these instructions and microscopic slides to the patient, a clinician can show interest in the specimens collected by the patient without having to disrupt the practice trying to manage specimens in disagreeable scenarios. It is of paramount importance that these patients feel seen and heard in order to establish the rapport necessary for treatment compliance. This is a simple, yet highly effective way, of doing so. It is also best not to mindlessly throw the specimen away after the microscopic examination because some patients can be very ego invested in keeping these specimens as “evidence.”

## Conclusion

Dermatology providers often find it difficult to treat DOP/DI. This is exacerbated by the fact that are few specialized psychodermatology clinics, even though there is evidence demonstrating that these clinics can offer cost-effective care.^23^ As stressed above, building rapport is the most important initial step when treating these patients because connecting with these patients is often the biggest challenge.^17^ Furthermore, pimozide is uniquely acceptable to this patient population given the lack of an US FDA psychiatric medication.^24^ However, with the right attitude, determination and commitment to continuing education, these patients can be some of the most grateful seen in your practice.

## Author contributions

N.B., J.K., J.H.: Participated in the expert interview.N.B.: Composed the article.J.K., J.H.: Revised the article and provided critical revisions for the final draft.

## Conflicts of interest

None.

## Funding

None.

## Study approval

N/A.
